# Familial hypercholesterolemia revealed by multiple xanthomas

**DOI:** 10.11604/pamj.2018.30.29.15719

**Published:** 2018-05-16

**Authors:** Nassiba Elouarradi, Nawal El Ansari

**Affiliations:** 1 Service of Endocrinology, Diabetology and Metabolic Diseases, University Hospital of Marrakech, Marrakech, Morocco

**Keywords:** Familial hypercholesterolemia, xanthomas, LDL

## Images in Medicine

Familial hypercholesterolemia (HF) is a rare pathology characterized by a major elevation of LDL associated with tendinous and subcutaneous xanthomas. We report the case of a 16-year-old patient, born from a first degree consanguineous marriage, with no particular pathological antecedents, the patient reports appearance for 9 years of cutaneous lesions described as a swellings in the posterior face of the 2 elbows and the anterior surface of the 2 knees, painless, firm, and gradually increasing in volume. The patient consulted only at the age of 16 years in front of the aesthetic discomfort. The biological assessment objectified a hypercholesterolemia: total Cholesterol: 4.44g/l. HDL: 0.64g/l. LDL: 3.71g/l. and Triglycerides: 0.67g/l. The exploration of cardiac repercussion was without particularities.The patient was treated with dietary and lifestyle measures and atorvastatin 80 mg with good clinical and biological evolution.

**Figure 1:**
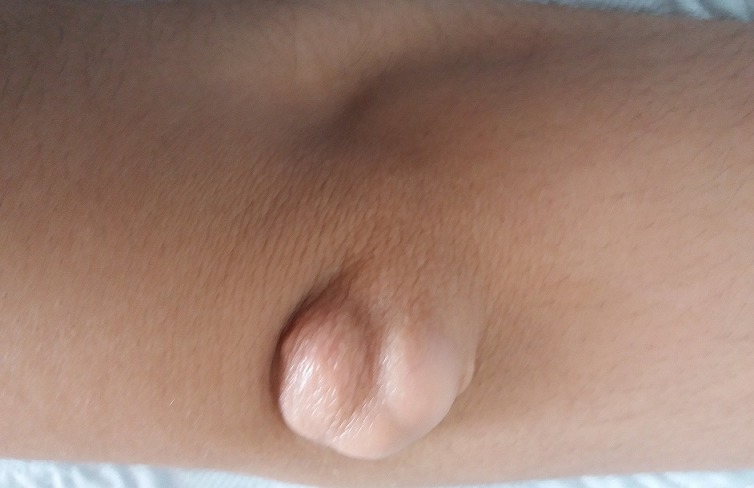
xanthoma on the posterior face of the elbow

